# Targeting ATG4 in Cancer Therapy

**DOI:** 10.3390/cancers11050649

**Published:** 2019-05-10

**Authors:** Yuanyuan Fu, Zhiying Huang, Liang Hong, Jia-Hong Lu, Du Feng, Xiao-Ming Yin, Min Li

**Affiliations:** 1 School of Pharmaceutical Sciences, Guangdong Provincial Key Laboratory of New Drug Design and Evaluation, Sun Yat-Sen University, Guangzhou 510006, China; fuyy6@mail2.sysu.edu.cn (Y.F.); hzhiying@mail.sysu.edu.cn (Z.H.); hongliang@mail.sysu.edu.cn (L.H.); 2 State Key Laboratory of Quality Research in Chinese Medicine, Institute of Chinese Medical Sciences, University of Macau, Macau, China; jiahonglu@umac.mo; 3 State Key Laboratory of Respiratory Disease, School of Basic Medical Sciences, Guangzhou Medical University, Guangzhou 511436, China; feng_du@foxmail.com; 4 Department of Pathology and Laboratory Medicine, Indiana University School of Medicine, Indianapolis, IN 46202, USA

**Keywords:** autophagy, ATG4, ATG4B, cancer therapy

## Abstract

Autophagy is a lysosome-mediated degradation pathway that enables the degradation and recycling of cytoplasmic components to sustain metabolic homoeostasis. Recently, autophagy has been reported to have an astonishing number of connections to cancer, as tumor cells require proficient autophagy in response to metabolic and therapeutic stresses to sustain cell proliferation. Autophagy-related gene 4 (ATG4) is essential for autophagy by affecting autophagosome formation through processing full-length microtubule-associated protein 1A/1B-light chain 3 (pro-LC3) and lipidated LC3. An increasing amount of evidence suggests that ATG4B expression is elevated in certain types of cancer, implying that ATG4B is a potential anticancer target. In this review, we address the central roles of ATG4B in the autophagy machinery and in targeted cancer therapy. Specifically, we discuss how pharmacologically inhibiting ATG4B can benefit cancer therapies.

## 1. Introduction

Autophagy is an evolutionarily self-catabolism process that degrades aged, defective or unnecessary cellular contents, using lysosomes to sustain cell metabolism and survival. Autophagy is typically divided into five stages: initiation, nucleation, maturation, fusion with the lysosome, and degradation [1]. The core autophagy machinery of cells is encoded by a set of highly conserved autophagy-related genes (ATGs), which were first discovered in yeast, and their orthologs have been well identified in mammalian cells. It is believed that targeting autophagy can be an important strategy to prevent or to treat many common human diseases, particularly cancer. 

**Figure 1:**
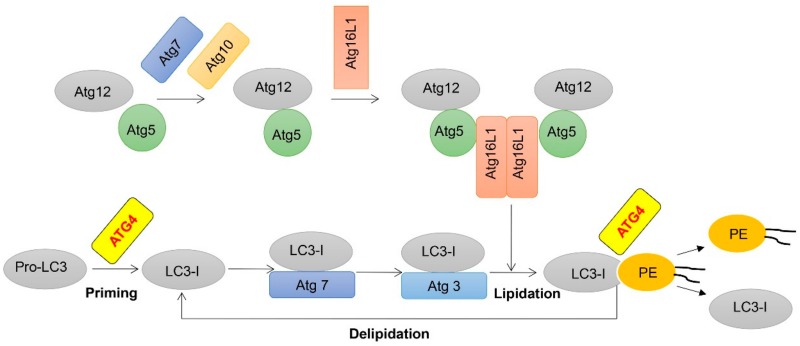
Schematic representation of two ubiquitination-like modifications essential for autophagy. Following translation, most pro-LC3 is cleaved into LC3-I by autophagy-related gene (ATG)4, LC3-I is activated by the E1-like molecule Atg7, transferred to the E2-like molecule Atg3 and finally conjugated to phospholipids with the assistance of the E3-like Atg12-Atg5-Atg16L1 complex. Next, membrane-localized LC3-II is also delipidated by ATG4 to recycle LC3-I.

Autophagosome biogenesis requires two ubiquitin-like conjugation systems: the Atg12-Atg5 and the Atg8-phosphatidylethanolamine (PE) system. ATG4 plays a key role in the regulation of the Atg8/microtubule-associated protein 1A/1B-light chain 3 (LC3) lipid conjugation system (Figure 1), which is required for the cleavage of pro-LC3 to the soluble form LC3-I and the deconjugation of the lipidated form of LC3 (LC3-PE, i.e., LC3-II) from autophagosome membranes or non-autophagosome structures to release LC3-I to facilitate autophagy process [2]. It has been reported that the delipidation process executed by ATG4B is important not only for the recycling of LC3-I, but also allowing the fusion between autophagosomes with lysosomes [3]. There are four ATG4 homologs in human genome: ATG4A, ATG4B, ATG4C and ATG4D. It has been assumed that these four homologs have both overlapping and unique functions. ATG4B is most proteolytically active toward both LC3 and GABA type A receptor-associated protein (GABARAP) subfamilies, while ATG4A is only effective for GABARAP subfamily proteins. On the contrary, ATG4C and ATG4D are almost inactive without any modifications [4]. Studies have shown that overexpression of an inactive mutant ATG4B^C74A^ or ATG4B^C74S^ causes defects in autophagosome closure and autophagic flux inhibition [5]. Our previous study has demonstrated that knockout of ATG4B in HeLa cells could completely inhibit the autophagy flux, even if other autophagy inducers or inhibitors were treated [6]. Autophagic activity was diminished in Atg4-deficient yeast [7], and mice lacking ATG4B showed reduced autophagy activity and a balance-related behavioral phenotype that is linked to inner ear developmental defects [8], while mice lacking ATG4C displayed a minimal impact on autophagy [9]. In addition, contrasting with other essential autophagy genes such as ATG5, ATG7 or VPS34, ATG4B and ATG4C-deficient mice presented a minor phenotype [8,9]. While ATG5-null mice were neonatal lethal [10], ATG7- and VPS34-deficient mice showed severe phenotypes in multiple tissues and exhibited decreased survival rates [11,12]. Thus, ATG4B is considered to be one of the most important ATGs in autophagy process since ATG4B plays an indispensable role in the Atg8-PE ubiquitin-like conjugation system. 

Autophagy exists in both physiologic and pathophysiologic conditions, and dysregulation of autophagy has been implicated in the development of many diseases, including Alzheimer’s disease [13,14], microorganism infection [15], immune homeostasis [16], Pompe disease [17] and cancer [18,19]. In a large number of normal human cells, autophagy is slightly activated at a basal level to maintain the physiological homeostasis [20], but under some pathophysiologic conditions the state of autophagy may alter significantly. Autophagy plays dual roles in cancer biology by suppressing tumorigenesis but promoting the survival of advanced tumors. Abundant studies have shown that autophagy inhibition can synergize with chemotherapy to enhance the efficacy of anticancer therapies [21,22,23,24]. In addition, genetic knockdown of autophagy-related genes is also an effective way to enhance the efficacy of anti-cancer drugs [25]. So, there is a growing need to discover and develop potent autophagic inhibitors. Currently, autophagy inhibitors used in cancer therapy are mainly lysosomal inhibitors, such as chloroquine (CQ) and CQ derivatives [26,27,28]. The next generation of lysosomal inhibitors including Lys05 can effectively inhibit autophagy and impair melanoma growth in preclinical mouse models [29]. However, these lysosomal inhibitors may be autophagy independent and simply de-acidifying the lysosomes. Therefore, the development of autophagy-specific inhibitors is a new therapeutic strategy for anticancer therapy. Currently, inhibitors against other autophagy targets such as the class III PI3K (VPS34) [30], ULK1 [30] and ATG4B [31] have also been reported. Growing evidence has shown that ATG4B is involved in certain physiological and pathological tumor microenvironments through affecting the autophagy process [31,32,33,34,35], and ATG4B has gained much attention to be a potential drug target for cancer therapy. In this review, we mainly focus on the pivotal role of ATG4 in autophagy, the progress of research on ATG4 inhibitors and the prospect of the cancer therapy by targeting ATG4 inhibition. 

## 2. A Mechanistic Understanding of ATG4 in Autophagy

In eukaryotes, the hallmark of autophagy is the formation of double-membrane autophagosomes, which are decorated with LC3B, a protein essential for their fusion with lysosomes and therefore widely accepted as a marker for autophagy activity assessment. LC3B is present on both sides of the autophagosomes [36], and it also functions as an anchoring point for cargo receptors [37]. Mammalian genomes encode at least seven Atg8 homologs being divided into two subfamilies: LC3 (MAP1LC3A, B, B2 and C) and GABARAP (GABARAP, GABARAPL1 and GABARAPL2/GATE-16) [38]. These homologs share a high sequence similarity and have a conserved glycine residue in C-terminal. Among these homologs, LC3B is the most studied and best understood LC3/GABARAP family protein and is the main substrate of the cysteine protease ATG4B.

During the autophagy process, Atg8-family proteins can undergo two distinct proteolytic steps. Firstly, ATG4B cleaves pro-LC3 to expose a glycine residue at site 120 near the C-terminus, followed by the sequential steps leading to lipid conjugation, including the interaction with Atg7 (E1-like enzyme), Atg3 (E2-like enzyme) and the Atg12-Atg5-Atg16L complex (E3-like ligase enzyme) to form membrane-bound Atg8/LC3-PE [39,40]. In the course of the ubiquitination-like reaction, ATG4B and the LC3 homologues should associate to achieve the cleavage step, but once the cleavage is completed, the LC3-I should separate from ATG4B for the subsequent interaction with Atg7. There is a possibility that an unknown factor is vital for separating LC3 from ATG4B, and ATG4B overexpression might be one limitation. The LC3-PE conjugation is required for autophagosomes expansion and maturation. Secondly, following autophagosome closure, ATG4B is reactivated to delipidate LC3 off from the membrane at the bond between glycine residue and PE [41]. The autophagosomes then fuse with lysosomes and cause degradation of the engulfed contents by lysosomal enzymes. 

It has been reported that the first step of the processing is very fast and even a small amount of ATG4B is sufficient for this natural processing work [42]. Hence, the unprocessed forms of Atg8-family proteins cannot be observed in both normal yeast and mammalian cells. Studies from many groups have suggested that knockdown of ATG4B could not ordinarily cause the accumulation of full length Atg8-family proteins, we can only detect the processed form of Atg8-family proteins, and under this situation, the expression of lipidated Atg8 is increased, perhaps because limited delipidation activity [32,43,44]. Studies have shown that pro-LC3B could accumulate only when the ATG4B expression is eliminated totally by genetic knockout [6,7,45]. It is noted that other ATG4 paralogs are much slower than ATG4B in the cleavage of LC3. An in vitro study has suggested that ATG4B is catalytically 1500-fold more efficient than ATG4A for LC3B [4], so other ATG4s could not compensate the function of ATG4B. However, a most recent research showed that ATG4B was required for LC3B lipidation but not for GABARAPL1 and GABARAPL2 lipidation. In addition, ATG4C and ATG4D both contribute to GABARAP isoform cleavage [46]. 

In contrast to the lipidation step, the mechanism of ATG4-mediated delipidation of Atg8-PE remains elusive. This process cannot be fast and may be regulated by other proteins or by some post-translational modifications. Studies have shown that during starvation-induced autophagy the phagophore growth lasts at least 10 to 20 min in order to support autophagosome extension and maturation [47,48]. Thus, the delipidation activity of ATG4 must be regulated someway to delay the deconjugation of Atg8-PE until the final stage of autophagosome maturation. Undoubtedly, the delipidation activity of ATG4 is important for the autophagy flux. Delipidation is for the recycle of Atg8-PE not only on autophagosomes but also on non-autophagosomal membranes to circulate the autophagy flux [7]. 

## 3. The Structure and Post-Translational Modifications of ATG4

**Figure 2:**
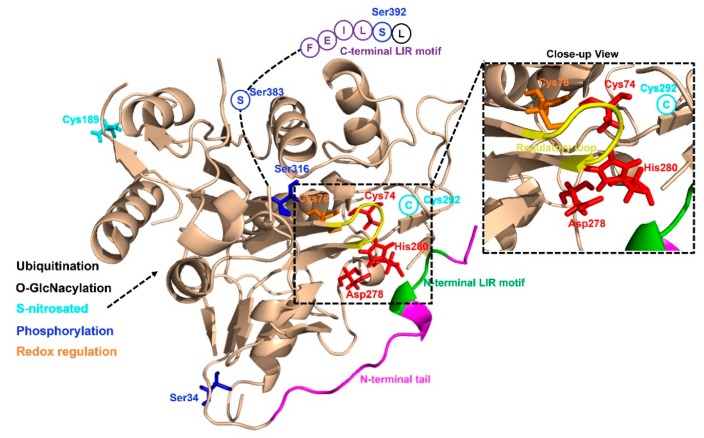
Structural basis of ATG4B and its post-translational modifications. S-nitrosated sites are colored cyan, and phosphorylation sites are colored blue, Redox regulation sites are colored orange, respectively.

As yet, little is known about the precise mechanisms that regulate ATG4 activity in cells. Human ATG4B (a member of C54 peptidase) has 393 amino acids and contains several structural features of cysteine proteinases (Figure 2). The catalytic triad of ATG4B consists of Cys74, Asp278 and His280, and studies have shown that mutations of any of these sites lead to a complete loss of ATG4B activity [49]. The crystal structures of human ATG4B (PDB ID: 2CY7 and 2D1I) at 1.9- and 2.0-Å resolution reveal that the papain-like fold and the small α/β-fold domain of ATG4B contribute to the interaction between ATG4B and Atg8 homologues [49,50]. Intriguingly, site Cys74 of human free ATG4B is auto-inhibited by an inhibitory loop (residues 259–262), suggesting that ATG4B may undergo conformational changes upon forming complex with LC3B. The published crystal structure for human ATG4B and LC3B complex (PDB ID: 2ZZP), a C-terminal deletion mutant ATG4B (1–354) and full-length LC3B, reveals that the N-terminal tail of ATG4B (residues 1–24) and the regulatory loop (residues 259–262) undergo a conformational change when ATG4B interacts with LC3B [2]. A recent work has shown that ATG4B contains two putative LC3-interacting region (LIR) motifs, whereas only the C-terminal LIR motif (FEIL), which is located in the highly flexible C-terminal residues (388 to 391) of ATG4B, could affect the binding and efficient cleavage of Atg8-family proteins [51]. The structure of ATG4A has also been resolved (PDB ID: 2P82), and through homology modeling the three-dimensional structures of ATG4C and ATG4D are available [52]. Different from ATG4B and ATG4A, ATG4C and ATG4D have extended N-termini, containing canonical DEVD caspase sites. Similar to ATG4B, the N-terminus of free ATG4C and ATG4D were shown to occlude the catalytic site of these two enzymes, and the extended N-termini making it difficult for the substrate to access [53]. Besides, ATG4D contains a putative Bcl-2 homology 3 (BH3) domain towards the end of the molecule. Collectively, further studies are needed to explain the complexity of the interaction between ATG4 and Atg8-family proteins for a better understanding of the regulation of autophagosome biogenesis. 

Recent studies have demonstrated that some post-translational modifications could regulate ATG4B activity (Figure 2). For example, Scherz-Shouval et al. have illustrated that human ATG4A and ATG4B are both regulated by reactive oxygen species (ROS) at the site of cysteine 81 and cysteine 78, respectively, and that this redox regulation of ATG4 plays a key role in the rapid activation and inactivation of these proteases [42]. Similarly, the investigation of the redox regulation of yeast ATG4 unraveled that cysteines 338 and 394 of ATG4 could form a disulfide bond to regulate ATG4 activity and this regulation is reversible. Besides the researchers also found that the reduced form of yeast ATG4 is its active form, suggesting that ATG4 can be redox regulated [54]. Further studies are still required to investigate the exact redox regulatory mechanisms affecting ATG4. Moreover, ATG4B was found to be S-nitrosated at cysteine 189 and cysteine 292 in the hippocampus of GK rats and in neuronal cells cultured in medium containing high levels of glucose. ATG4B S-nitrosation was considered to be associated with decreased autophagy activity [55]. In addition, O-GlcNacylation of ATG4B was increased in metabolic stress conditions and the proteolytic activity of ATG4B for LC3 was increased under PugNAc-treated cells [56]. 

Moreover, it has been shown that ATG4B can be phosphorylated at serine 34 [57], serine 383, and serine 392 from several large-scale phosphoproteomics studies [58,59,60]. Yang et al. had first shown that phosphorylation of ATG4B at serine 383 and serine 392 by as yet unidentified kinases could increase its delipidation activity of LC3B [61]. Huang et al. described that radiation induced MST4 kinase expression and MST4 kinase phosphorylated ATG4B at serine 383, which was critical in stimulating ATG4B activity and autophagic flux [62]. Ni et al. showed that the phosphorylation of ATG4B at serine 34 residue by AKT in hepatocellular carcinoma (HCC) cells had little effect on autophagic flux, but increased the Warburg effect of cancer cells [63]. Another study discovered that phosphorylation of human ATG4B at serine 316 by Unc-51 like autophagy activating kinase 1 (ULK1) could cause the inhibition of its catalytic activity in vitro and in vivo [64]. However, the mechanism of this regulatory phospho-switch between ULK1 and ATG4B is presently unclear. Remarkably, serine 307 of yeast ATG4 could be phosphorylated by Atg1 kinase to inhibit ATG4 proteolytic activity [65]. 

It was reported that ubiquitination also regulates ATG4B activity. The E3 ligase ring finger protein 5 (RNF5) could induce ATG4B ubiquitination and proteasome-mediated degradation to negatively regulate autophagy through inhibiting the processing of LC3B [66]. Taken together, the activity of ATG4B is regulated not only by translational control but also by multiple post-translational modifications, including redox regulation, S-nitrosation, O-GlcNacylation, phosphorylation and ubiquitination.

## 4. ATG4 and Cancer

It is thought that autophagy plays a context-dependent role in cancer by preventing cancer development at the initiation stages, but improving the survival of cancer cells at the later stages from stressful conditions, such as hypoxia, nutrient deprivation and therapeutic damages [67,68,69,70]. Many studies have shown that autophagy contributes to tumors by supplying ATP to maintain cellular biosynthesis and survival. Thus inhibition of autophagy could be an effective way for cancer control [71,72,73]. The cysteine protease ATG4B is a potential anti-cancer target due to its roles in the regulation of autophagy. Studies have shown that knockout of ATG4B or expression of the catalytically negative mutant ATG4B^C74A^ could suppress autophagy as measured by a higher level of sequestosome 1 (SQSTM1) and the lack of enclosed autophagosomes [5,6,8]. Surprisingly, overexpression of the active ATG4B also arrests autophagy by the same measures [6], suggesting an auto-inhibitory role for ATG4B in autophagic pathway. Accordingly, a suitable level of ATG4B is essential to maintain a balance between the soluble and lipidated forms of LC3B for an appropriate autophagic activity. 

The role of ATG4B in cancer has been recently reported [31,33,34,74]. For example, tumor cells of colorectal cancer patients had significantly higher ATG4B expression level than adjacent normal cells, suggesting that ATG4B plays a positive role in colorectal cancer development although the enhancement for cell proliferation by ATG4B is independent of autophagy [74]. In addition, osteosarcoma Saos-2 cells lacking ATG4B failed to grow as a xenograft in nude mice due to a defective in autophagy activity [65].The expression of ATG4B was increased significantly in human epidermal growth factor receptor 2 (HER2) positive breast cancer cells and ATG4B was required for these cells to survive under stressful conditions, implying that this subtype of breast cancers is suitable for employing ATG4B inhibition strategies [32]. In prostate cancer cells, inhibition of ATG4B with a dominant negative form ATG4B^C74A^ resulted in a cell line-specific susceptibility to chemotherapy and radiotherapy [34]. Similarly, overexpression of the dominant negative mutant ATG4B^C74A^ in Huh7 cells could decrease the cell viability [75]. In chronic myeloid leukemia (CML) stem/progenitor cells, ATG4B was highly expressed and knockdown of ATG4B suppressed autophagy, reduced the survival of these cancer cells and sensitized them to chemotherapy treatment [33]. Inhibition of ATG4B in a subset of glioblastoma that has increased ATG4B phosphorylation resulted in the suppression of the tumors in animal models [62]. Finally, in human lung cancer cells, ATG4B directly interacted with soluble carrier family 27 member 4 (SLC27A4) to form a SLC27A4/ATG4B complex, and knockdown of SLC27A4 could decrease the ATG4B level, thus enhancing the therapeutic efficiency of chemotherapeutic drugs [76].

Notably, several studies have also indicated the role of ATG4A in cancer. For example, it is reported that women carrying a variant allele of ATG4A faced lower risk of ovarian cancer [77]. In another study, a high degree of methylation of ATG4A was observed in ovarian cancer patients, implying that ATG4A may have clinical significance in ovarian cancer [78]. In addition, ATG4A has also been reported to be related to the survival of breast cancer stem cells [79], and the risk of cervical cancer and lung cancer [80,81]. 

ATG4C and ATG4D have also been implicated in cancer biology. Knockout of ATG4C increased the susceptibility to develop fibrosarcomas induced by methylcholanthrene in mice, suggesting that ATG4C may play a tumor-suppressor role [9]. In contrast, ATG4C was also reported to play a tumorigenic role in breast cancer [82]. Such results indicate that the role of ATG4C in cancer development may be context dependent. Furthermore, studies have shown that inhibition or silencing of ATG4D could sensitize cancer cells to chemotherapeutic drugs [53,83,84]. On the other hand, ATG4D was considered as a tumor suppressor in colorectal carcinogenesis since reduced ATG4D expression was observed in adjacent normal cells [85]. Collectively, it is necessary to clarify the precise mechanisms of ATG4 in cancer development and targeted cancer therapy in order to develop novel regulators for anticancer therapies.

## 5. Pharmacological Targeting of ATG4B in Cancer Therapy 

Several preclinical studies have shown that induction of autophagy can promote cancer cell survival in resistance to cancer treatment and that autophagy inhibition through genetic or pharmacological means is an effective way to overcome treatment resistance [86,87,88]. Nowadays, CQ and its derivatives are the most broadly applied autophagy inhibitors, which have been used in preclinical studies or clinical trials for various cancers [89,90,91,92]. More than 30 clinical trials employing CQ or hydroxychloroquine (HCQ) have been developed around the world (http://clinicaltrials.gov/). However, such anti-lysosomal agents may not just inhibit autophagy to exert their anticancer action, CQ and its derivatives may also affect other pathways to alter cellular metabolism and cause unfavorable side effects [91,93]. In addition to these lysosome inhibitors, other autophagy related targets such as ULK1, VPS34, BECN 1 and ATG4B have also been targeted by small molecule inhibition for cancer chemotherapy. An important unanswered question that is raised with autophagy inhibitors is whether it is better to target early steps in the autophagy pathway to stop the formation of autophagosomes, or to block the fusion between autophagosomes and lysosomes, or to block the degradation of autolysosomes. Since autophagosomal structures were reported to serve as scaffolds to induce apoptosis and necroptosis [94,95]. It might be better to block degradation of autophagosomes with a lysosome inhibitor or ATG4B inhibitor rather than ULK1, VPS34 and BECN 1 inhibitors. Besides, BECN1/VPS34 complex have other autophagy-independent functions, so the suppression of BECN/VPS34 might also alter other cellular processes. 

**Figure 3:**
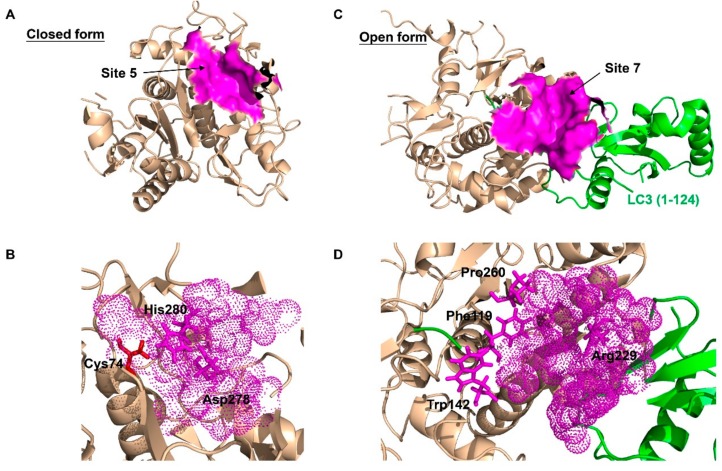
The best predicted-docking pockets of the closed form and open form of ATG4B structure. (**A**) Crystal structure of the closed form of ATG4B protein (PDB ID: 2CY7) with labeled pocket site 5. Site 5 contains Thr10, Leu11, Ala14, Asn261, Ser262, His264, Tyr276, Asp278, His280 and Cys306. Site 5 is shown with surface and colored magenta. (**B**) Close-up view of site 5, where site 5 is shown with dots and colored magenta. The active site amino acids Cys74 (red), Asp278 (magenta) and His280 (magenta) are shown with sticks. Asp278 and His280 are together within the pocket surface that is suitable for molecular docking. (**C**) Crystal structure of the open form of ATG4B (PDB ID: 2Z0E) with labeled pocket site 7. Site 7 contains Trp142, Arg229, Leu232, Thr233, Pro260, Asp314, Pro315 and Ser316 of ATG4B, and Tyr38, Gly40, Glu41, Lys42, Gln43, Ala114, Ser115, Gln116, Glu117, Thr118 and Phe119 of LC3B. Site 7 is shown with surface and colored magenta. (**D**) Close-up view of site 7, where site 7 is shown with dots and colored magenta. Residues Trp142, Arg229 and Pro260 of ATG4B, and Phe119 of LC3B, which are clustered within the pocket surface and related to the interaction, are shown with sticks. The best docking pockets were defined by MOE2010 and all structure models were generated using PyMOL.

ATG4B is considered as an alternative strategy to anti-lysosomal therapy due to its effect at a different stage of autophagy. Thus, drugs targeting ATG4B are more specific to the autophagy process and could have a more critical role in modulating autophagy. As such ATG4B has been considered as a potential therapeutic target for cancers. As mentioned above, ATG4B could act as a pro-tumorigenic gene in cancers expressing it at a high level, including osteosarcoma, CML, HER2-positive breast and colorectal tumor cells. Recently, a number of small molecule ATG4B inhibitors have been discovered, which showed effective inhibition effects on the activity of ATG4B in vitro and/or in vivo. Correspondingly, plenty of methods have been developed for screening ATG4 inhibitors in vitro, including the classical SDS-PAGE-based assay [36,96], the high-throughput method using LC3B-PLA2 fluorogenic peptide [97,98], the FRET-based assay and the TR-FRET-based assay [99,100,101]. Recently, computer-aided virtual screening has been carried out by a number of groups for ATG4B regulators [6,31,44,102,103], since the crystal structures of ATG4B become available. which include the closed form of ATG4B (PDB ID: 2CY7/2D1I), and the open form of ATG4B (PDB ID: 2Z0D/2Z0E) and its inactive conformation (PDB ID: 2ZZP). By finding the suitable pockets from closed and open ATG4B structures (Figure 3), thousands of small molecular compounds could be screened by molecular docking in a very short time. Besides, methods to measure ATG4 activity ex vivo have also been developed, such as the conventional exogenous overexpression of ACTB-LC3B-dNGLUC or LC3-GST in cells [6,31,104], the FITC-labeled ATG4-substrate peptides [105], or examining the fluorescence ratio between AU4S and AU4R [106]. 

**Figure 4:**
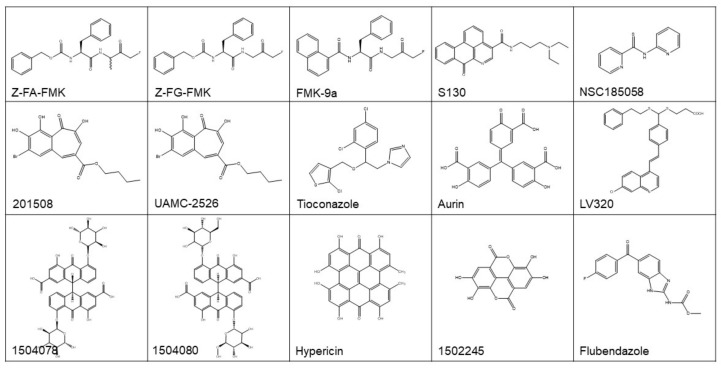
The structure of reported ATG4B regulators. See the text and Table 1 for more information.

With these methods, a number of promising ATG4 inhibitors have been developed and identified (Figure 4 and Table 1). For example, Shu et al. identified 4 ATG4B inhibitors from 3280 bioactive compounds (1280 from Lopac™ and 2000 from Spectrum™ library) with IC_50_ < 10 µM using the LC3B-PLA2 reporter assay, yielding a hit rate of 0.23% and 0.70%, respectively [107]. Subsequently, this group of researchers screened a pool of FDA-approved drugs for ATG4 inhibitors employing virtual docking and molecular dynamics simulations. By using LC3B-PLA2 reporter assay, tioconazole with an IC_50_ of 1.8 µM was identified as a more potent ATG4B inhibitor. Further studies showed that tioconazole diminishes autophagic flux and sensitizes cancer cells to chemotherapeutic drugs [44]. 

Xu et al. reported the identification of Z-FA-FMK, a covalent ATG4B inhibitor found from a Roche focus library of 57,000 compounds using the TR-FRET assay [100]. The optimization of the structure of Z-FA-FMK led to the discovery of Z-FG-FMK and a series of potent FMK-based ATG4B inhibitors. Among these compounds, FMK-9a showed the best inhibition efficiency on ATG4B in vitro, with an IC_50_ of 80 nM in the TR-FRET based assay [101]. On the basis of these researches, Chu et al. showed that FMK-9a could inhibit the activity of ATG4B both in vitro and in cells. Paradoxically, FMK-9a could initiate the onset of autophagy, suggesting that FMK-9a may have other targets in autophagy process [108]. 

In a study performing FRET-based assays, hypericin and aurin tricarboxylic acid were found to have an IC_50_ of 30 and 8.8 µM, respectively, for ATG4B [109]. In another study performing a combined computer-aided drug screening and biochemical analysis, LV-320 was found to be an ATG4B inhibitor with an IC_50_ of 24.5 µM [103]. LV-320 suppressed autophagic flux and was active in vivo with an excellent pharmacokinetic profile. Kurdi et al. found that compounds with a benzotropolone core structure have ATG4B inhibiting potential. Then, 27 derivatives of benzotropolones were synthesized and screened with an inhibition rate for recombinant ATG4B ranged from 3 to 82%. Among them, compound UAMC-2526 abolished autophagy and improved the sensitivity of HT29 colorectal tumor to chemotherapy [110].

The study by Akin et al. was the first one performing in silico docking assays to screen for ATG4B inhibitors [31]. NSC185058 was identified as an ATG4B inhibitor from a NCI library, which had an IC_50_ of 51 µM based on an in vitro LC3-GST cleavage assay. NSC185058 was reported to inhibit autophagy and to suppress the growth of Saos-2 osteosarcoma tumors. NSC185058 was also found to markedly attenuate autophagy and enhance the anti-tumor activity of radiation therapy in glioblastoma in xenograft models [62]. In our previous study, we discovered a novel ATG4B antagonist with an IC_50_ of 3.2 µM from a noncommercial library by integrated in silico screening and in vitro assays [6]. This compound, named as S130, inhibited the autophagy flux and arrested the growth of colorectal xenograft models, this study advanced our knowledge of the association of ATG4B and colorectal cancers. Altogether, these data demonstrate that ATG4B could be a suitable therapeutic target for treating cancers. 

## 6. Prospect of the Cancer Therapy Targeting ATG4B Inhibition

Due to the key role of ATG4B in autophagy process, ATG4B has attracted a great level of interest in the field of autophagy research and ATG4B inhibitors have been proposed as a potentially therapeutic approach for not only cancers but also other human diseases. In most normal tissues, the expression level of ATG4 homologues is low, with the highest protein expression level of ATG4s being detected in skeletal muscle, followed by brain, heart, liver, pancreas, and testes [111]. As mentioned above, a high expression level of ATG4B seems to be connected with the progression of tumor as well as with cancer therapy resistance. Apart from ATG4B, other mammalian ATG4 homologues may also be potential therapeutic targets for cancer, particularly ATG4D, which plays a notable role in apoptosis [53,112,113]. However, no published data have shown the prognostic value of any ATG4 members in cancers [114].

Paradoxically, both agonists and antagonists of ATG4B could have therapeutic significance in cancer. Most of the reported regulators of ATG4B were inhibitors, only one compound named Flubendazole was indicated as an ATG4B agonist, which could induce autophagic cell death in MDA-MB-231 cells [102]. Most of the discovered ATG4B inhibitors were only verified in vitro without further confirmation and in vivo testing. Moreover, ATG4B inhibitors with diverse structures were able to inhibit tumor growth in vivo [6,31,44], implying that these compounds may also affect targets other than ATG4B in cells. 

The in vivo inhibitory efficacy of ATG4B modulators has not been well established. FMK-9a, with an IC_50_ of 80 nM, is the most potent ATG4B inhibitor so far reported, but it could neither reduce the survival rate of cancer cells nor improve the anti-tumor effect of chemotherapeutic drugs within a reasonable concentration range (Fu and Li, unpublished results), suggesting that the antitumor effect of ATG4B inhibitors may not necessarily be correlated with the inhibition of ATG4B enzymatic activity as measured by in vitro assays. One study has shown that the growth arrest induced by knockdown of ATG4B in human colorectal cancer cells was independent of the autophagic flux, suggesting that ATG4B could have additional functions independent of autophagy [74]. Thus, it seems that the application of ATG4B inhibitors to modulate autophagy and cancer cell growth may have to be context-dependent.

In summary, while substantial progress has been made in the clarification of mechanism and function of ATG4 in autophagy and cancer therapy, more work remains to be done. As an appropriate level of ATG4 is essential for autophagy flux, it is necessary to determine how ATG4 is spatially and temporally regulated in cells. In addition, since ATG4 drives the cleavage of Atg8-family proteins at the beginning and the ending of autophagosome formation, it is necessary to further elucidate which process or if perhaps both processes may need to be targeted in order to exert the best anti-cancer effect. Moreover, ATG4-family proteins have four homologues and thus the reported ATG4B inhibitors could also target other ATG4s, it is necessary to understand whether specific ATG4B inhibitors or non-selective ATG4 inhibitors have better anti-cancer effects. It is also necessary to co-crystallize ATG4B with its inhibitors to better elucidate the biological roles of ATG4B in cells, which will in turn enable the design of more effective ATG4B inhibitors. Thus, more specific ATG4 regulators are needed for the study of the complicated role of ATG4 in autophagy and cancer cell biology. In conclusion, ATG4 plays a crucial role as a potential target in cancer biology, but more extensive studies are required to comprehend this unique protease.

## 7. Conclusions

In this review, we summarized how ATG4-family proteases are involved in the autophagy process, their recent structural and functional studies, their roles in cancer biology and targeted cancer therapy. Specifically, we highlighted recent advances in our knowledge of ATG4 inhibition as an alternative strategy for autophagy inhibition. While multiple drugs could inhibit ATG4 activity, most lack specificity and anti-tumor activity. Thus, more potent and selective agents targeting ATG4 are needed and represent a high priority for cancer drug development.

## Figures and Tables

**Table 1:** List of reported ATG4B regulators.

Name/Compound ID	IC_50_	Methods	Effect on ATG4B	Effect on Autophagy	Effect on Cancer	Ref.
201508	2.3 µM	LC3B-PLA2 assay	Inhibiting	N.R.	N.R.	[107]
1502245	3.0 µM	LC3B-PLA2 assay	Inhibiting	N.R.	N.R.	[107]
1504078	1.7 µM	LC3B-PLA2 assay	Inhibiting	N.R.	N.R.	[107]
1504080	1.1 µM	LC3B-PLA2 assay	Inhibiting	N.R.	N.R.	[107]
Tioconazole	1.8 µM	LC3B-PLA2 assay	Inhibiting	Inhibiting	Enhancing Dox-induced cytotoxicity in colorectal cancer	[44]
Z-FA-FMK	14.8 µM	TR-FRET assay	Inhibiting	N.R.	N.R.	[100]
Z-FG-FMK	1.13 µM	TR-FRET assay	Inhibiting	N.R.	N.R.	[100]
FMK-9a	80 nM	TR-FRET assay	Inhibiting	Inducing	Having no effect on the survival of HeLa cells	[101,108]
Hypericin	30 µM	FRET assay	Inhibiting	N.R.	N.R.	[109]
Aurin	8.8 µM	FRET assay	Inhibiting	N.R.	N.R.	[109]
LV-320	24.5 µM	Fluorescent peptide substrate assay	Inhibiting	Inhibiting	N.R.	[103]
UAMC-2526	N.R.	LC3-GST cleavage assay	Inhibiting	Inhibiting	Enhancing Oxaliplatin-induced cytotoxicity in colorectal cancer	[110]
NSC185058	51 µM	In silico screening, LC3-GST cleavage assay	Inhibiting	Inhibiting	Suppressing the development ofSao-2 cells; enhancing the anti-glioblastoma activity of radiation therapy	[31,62]
S130	3.2 µM	In silico screening, FRET assay	Inhibiting	Inhibiting	Arresting the growth of colorectal cancer	[6]
Flubendazole	N.R.	In silico analysis	Inducing	Inducing	Inducing autophagic cell death in MDA-MB-231 cells	[102]

N.R.: not reported.
